# Basicity and Electrolyte Composition Dependent Stability of Ni‐Fe‐S and Ni‐Mo Electrodes during Water Splitting

**DOI:** 10.1002/cphc.201901219

**Published:** 2020-02-11

**Authors:** Jochem H. J. Wijten, Iván Garcia‐Torregrosa, Eva A. Dijkman, Bert M. Weckhuysen

**Affiliations:** ^1^ Inorganic Chemistry and Catalysis Debye Institute for Nanomaterials Research Universiteitsweg 99 Utrecht The Netherlands

**Keywords:** catalyst stability, hydrogen, Ni−Fe−S, solar fuels, water splitting

## Abstract

Non‐noble metal electro‐catalysts for water splitting are highly desired when we are moving towards a society where green electrons are becoming abundantly available, offering clear prospects to make our society more sustainable. In this work, Ni−Fe−S is reported as a high performing anode material for the water splitting reaction, operating at low overpotentials and showing high apparent stability. Furthermore, Ni−Mo electrodes are developed on metallic foam substrates and optimized in terms of their performance. The Ni−Fe−S material as anode, combined and integrated with Ni−Mo as cathode in a cell configuration, splits water at 10 mA cm^−2^ and a potential of 1.55 V. Similar to previous reports, we confirm that Mo leaches from Ni−Mo/Ni foam electrodes. Cycling tests and ICP‐AES measurements show that the stability of Ni−Fe−S is apparent, and that in reality S is leaching from the material as was already suggested in literature. We expand on this knowledge and show that the leaching of S is dependent on both pH and the cation used during electrocatalysis. Furthermore, we find that applying an oxidative potential is in truth stabilizing towards S and that the alkalinity causes leaching. S was furthermore mobile and found to segregate towards the surface. Finally, using too low pH values (11 and lower) result in the passivating hydroxide metal layers being destroyed and the Ni−Fe−S dissolving completely.

## Graphical abstract

**Please don't leach**! Non‐noble metal electro‐catalysts for water splitting are highly desired to move towards a more sustainable society. Ni−Fe−S as a high‐performance material combined with Ni−Mo splits water at 10 mA/cm^2^ and a potential of 1.55 V. Durability tests show that S is leaching from the material. We expand on this knowledge and show that the leaching and segregation of S is dependent on both pH and the cation used during electrocatalysis.

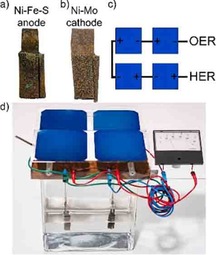

## 1 Introduction

One of the targets described in literature for solar‐driven water splitting cells to achieve 10 % solar‐to‐hydrogen (STH) efficiency is obtaining a combination of the hydrogen evolution reaction (HER) and the oxygen evolution reaction (OER) electrocatalysts, which operate at 10 mA/cm^2^ (geometric surface area) with a total overpotential (η_10_) lower than 450 mV.^1^, ^2^, ^3^, ^4^ 10 mA/cm^2^ is described as the current density output, which is typically found for solar driven water electrolyzers at 1 sun illumination.^1^

Importantly, the catalysts should maintain this low overpotential over an extended period of time to be viable to be used in water electrolyzers.^1^ Furthermore, it is desirable to produce catalysts based on earth‐abundant materials. For example, Pt is the best known mono‐metallic HER electrocatalyst,^1^ however, its scarcity and cost render it unsuitable for large‐scale water splitting applications. Based on literature one of the best performing HER electrocatalysts is Ni−Mo, which approaches the activity of Pt‐based catalysts.^1^, ^5^, ^6^ Whereas for OER, Ni‐S^7^, ^8^, ^9^, ^10^, ^11^ and Ni‐Fe^1^, ^12^ have been found as good electrocatalysts, which usually have an overpotential at about 300–400 mV at 10 mA/cm^2^. These materials still get trumped by Ir and Ru oxide catalysts; the latter of which reaches overpotentials of only 290 mV at 10 mA/cm^2^.^1^, ^7^, ^13^, ^14^ Ni−Fe−S is recently studied as a promising water splitting anode material reaching lower reported overpotential values for OER down to 65 mV.^15^, ^16^, ^17^, ^18^, ^19^ It should be noted that this low overpotential was, in part, due to the use of high surface area substrates (Ni foam).^15^ Furthermore, Ni−Fe−S can be used in seawater based electrolytes without forming chlorine.^20^ Ni−Fe−S can be easily formed through hydrothermal treatment^7^, ^9^, ^11^ or electrodeposition.^21^, ^22^, ^23^

Literature is currently in disagreeing on the stability of Ni−Fe−S, however.^15^, ^16^, ^17^, ^18^, ^19^ Many state that the material is stable,^16^, ^17^, ^18^, ^19^ though despite the claims of stability an significant increase of about 150 mV is seen over 200 h of continuous operation.^16^ The described mode of instability is S destabilization in alkaline media.^15^ This mode of destabilization is also underlined by the fact that dissolving Na_2_S in the electrolyte stabilizes the material as it supplies a source of S^2−^.^19^

In this work, we combine HER and OER electrocatalysts operating at low overpotentials. We show that Ni−Mo as a HER electrocatalyst and Ni−Fe−S as a OER electrocatalyst can achieve a current density of 10 mA/cm^2^ with an overpotential of only 320 mV, easily reaching the described target of overpotentials below 450 mV.^1^, ^2^, ^3^, ^4^ This goal is achieved by combining large surface areas per cm^2^ electrode through the use of metal foams with electrocatalysts.^9^ The catalysts are formed with simple and cheap one‐step methods, being electrodeposition (Ni−Mo) and hydrothermal treatment (Ni−Fe−S).^7^, ^9^, ^11^ With scanning electron microscopy (SEM), energy dispersive spectroscopy (EDX) and X‐ray diffraction (XRD) we observe the desired phases. The performance is stable for a week in 1 M alkaline solutions. Gas chromatography (GC) is used to confirm the Faradaic efficiency. Material stability was studied in detail and we found that S leaches from the Ni−Fe−S material as a function of pH and electrolyte cations.

## 2 Results and Discussion

### 2.1 Ni‐Mo versus Ni‐Fe‐S Water Splitting

**Figure 1:**
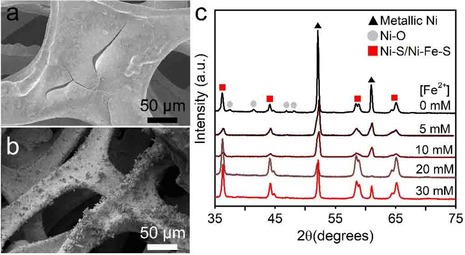
a) SEM of Ni−Mo coated on Ni foam. b) SEM of Ni−Fe−S coated on Ni foam from 5 mM Fe^2+^ solutions. c) XRD patterns of Ni−Fe−S on Ni foam synthesized at varying [Fe^2+^]. Peaks are indicated for metallic Ni (black triangles), NiO (grey circles) and Ni−S/Ni−Fe−S (red squares).

For Ni−Mo, homogeneous, amorphous coatings are formed via electrodeposition on high surface area (5400 m^2^/m^3^) Ni foams with a granular morphology containing several large cracks (Figure 1a), which is a typical morphology for these materials.^24^, ^25^, ^26^ With EDX an elemental distribution of 2 : 1 Ni : Mo was found; to minimize the contribution of the Ni foam substrate the Ni L‐edge peak is used to determine this ratio (Figure S1).^27^ Ni−Fe−S was prepared via a hydrothermal synthesis procedure (Figure S2).

Sharp nano pyramidal morphologies (Figure 1b) form during synthesis. They form as a result of the addition of Fe and grew more prominent with increasing Fe concentrations during the synthesis (Figure S3). The increase of surface area, that is a result of these morphologies forming, is likely the reason that Fe improves the performance of these materials.^28^, ^29^, ^30^, ^31^, ^32^ Fe is concentrated in the nano‐pyramids, as shown by EDX, while S and Ni are homogeneously distributed over the material (Figure S2 in the Supporting Information).

Using XRD (Figure 1c) it was found the formed Ni−Fe−S anodes are crystalline. The XRD peaks belonging to metallic Ni decrease in intensity with increasing Fe concentration, while XRD peaks ascribed to the Ni−Fe−S phase increase in intensity with increasing Fe concentration. This material forms the same crystal structure as Ni−S but with a slightly smaller unit cell as observed by the peaks being at slightly higher diffraction angles.^33^

**Figure 2:**
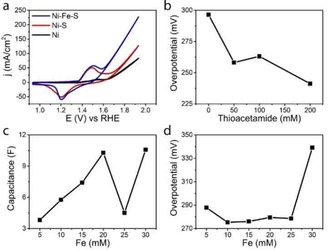
a) Comparison of CVs taken on bare Ni foam (black), Ni−S (red) and Ni−Fe−S (blue). b) The overpotential at 10 mA cm^−2^ with increasing thioacetamide concentrations (10 mM FeSO_4_). c) Increase of surface area, represented as the double layer capacitance slope, as a function of FeSO_4_ concentration during synthesis (50 mM thioacetamide). d) Behavior of the overpotential as a function of FeSO_4_ concentration (50 mM thioacetamide).

Various experimental parameters for the synthesis of Ni−Fe−S were varied to optimize the performance (Figure 2). Figure 2a shows that Ni−Fe−S outperforms Ni−S which in turn outperforms bare Ni foam. In each case a reversible peak is seen at 1.49 V (Ni^2+^→Ni^3+^) and 1.20 V (Ni^3+^→Ni^2+^). The observed overpotential towards OER for this material decreased with increasing S content, as shown by the amount of thioacetamide added (Figure 2b). Adding more thioacetamide than 210 mM resulted in significant embrittlement of the foam, however, and yielded electrodes not usable in electrochemistry. On the other hand, adding more Fe^2+^ into the electrode resulted in an increase in the capacitive slope found with double layer capacitance measurements (Figure 2c), suggesting an increase of surface area, agreeing with SEM (Figure S3). It had little influence on the overpotential up to 30 mM FeSO_4_ where a large loss of efficiency was observed (Figure 2d). This tells us that the S acts as a synergetic compound, increasing the activity whereas Fe serves to increase the surface area of the material.

**Figure 3:**
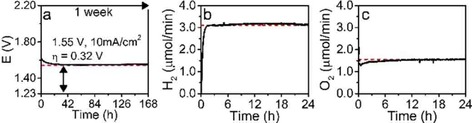
a) Chrono‐potentiometry of Ni (grey, dashed) and Ni−Mo (black, solid) at 10 mA cm^−2^ for 16 h using the materials as cathode with a Pt mesh as anode in a 2‐electrode cell configuration. b) Chrono‐potentiometry of Ni (grey, dashed) and Ni−Fe−S (black, solid) at 10 mA cm^−2^ for 16 h using the materials as anode with a Pt mesh as cathode. Both experiments are performed in 1 M NaOH in a 2‐electrode cell configuration.

The materials were then tested towards their respective reactions and, as shown in Figure 3, compared to bare, uncoated Ni foam. Ni−Mo vs Pt (Figure 3a) increased significantly in performance, reaching η_10_ values as low as 530 mV, compared to 850 mV when testing Ni vs Pt; an improvement of 320 mV. The Ni−Mo material has a short activation time of 2 h, which is a result of Mo leaching and a resulting increase of electrochemically active surface area.^34^ The anode: Ni−Fe−S, when tested vs Pt (Figure 3b) operates at an overpotential of 430 mV, compared to 610 mV for bare Ni vs Pt, improving by 180 mV. The tests with Pt as cathode have a significantly lower overpotential since Pt performs better towards the HER than the OER.^1^ In the case of Ni−Fe−S, there is a small increase in overpotential during the first hours of operation, being a result of oxidation to Ni(Fe,S)OOH surfaces.^35^, ^36^, ^37^

**Figure 4:**
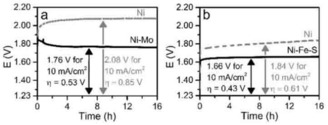
a) Chrono‐potentiometry in 1 M NaOH of Ni−Mo (HER) versus Ni−Fe−S (OER) and the corresponding stability over the course of 168 h in a 2‐electrode cell configuration, the red dashed line is set at 1.55 V. b) Hydrogen evolution in μmol/min (black) showing also the expected amount (Faradaic Efficiency of 101 %, red dashed) measured in the H‐cell. c) Oxygen evolution in μmol/min (black) showing also the expected amount (Faradaic Efficiency of 100 %, red dashed) measured in the H‐cell.

The OER and HER materials were combined in an integrated manner to test the performance of the combined setup for an entire week. As can be observed in Figure 4a the materials perform at a stable overpotential of 320 mV. The combination of these materials also shows an activation period like the separate materials, however, it is observable over a much longer timescale of 30 h. Faradaic Efficiency (FE) experiments show that we reach a value of 101.2 % for HER (Figure 4b) and a value of 99.7 % for OER (Figure 4c); both values are very close to the aimed value of 100 %. However, there was some small leaching of Mo and S from the respective samples, as observed by ICP‐AES (S.I. Table S.1).^34^ As can be observed, no significant amounts of Ni or Fe leach in any of the combinations of materials. S does leach somewhat from the Ni−Fe−S material, most significant in the first 16 h of electrolysis, but continues for longer timescales to 12 μmol after 1 week. Mo also leaches, as was previously found,^34^ and it leaches predominantly in the first hours of operation since no increase in leaching is found after 1 week of operation compared to 16 h.

In Figure S4 SEM is shown of Ni−Mo/Ni foam and Ni−Fe−S/Ni foam after the test of a week. As can be seen the main surface of the material does not change noticeably. The small particulate matter that is present prior to catalysis has mostly disappeared as was found to consist mostly of Fe with EDX, which explains the observed Fe in solution with ICP‐AES.

**Figure 5:**
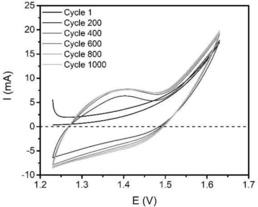
Cyclic voltammograms of continuous cycling for 1000 cycles of Ni−Mo vs Ni−Fe−S. Cycles are shown every 200 cycles. A large change was seen in the first few cycles, where the Ni^2+^→Ni^3+^ peak became more prominent which was fairly stable after. Nevertheless a slow increase of this peak as well as the OER current was observed over the course of cycling.

The leaching of S and Mo at a continuous current led us to do a stability test via cyclic voltammetry, measuring 1000 cycles at a scan rate of 50 mV/s to simulate current intermittency at a sped‐up rate. As can be seen in Figure 5 there is an obvious difference between the initial cycle and later cycles where the Ni^2+^→Ni^3+^ peak at 1.4 V grew significantly, after which slow growth continued during the cycling. The OER current increased slowly likewise but was predominantly stable. This shows us that though the material is clearly unstable it has a stable performance.

### 2.2 Electrolyte Effects on Ni‐Fe‐S Stability

**Figure 6:**
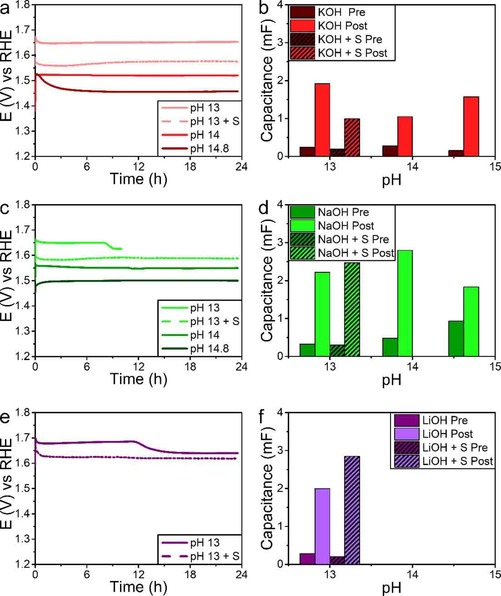
a) Chrono‐potentiometry of Ni−Fe−S/Ni foam at 10 mA cm^−2^ operated in KOH electrolytes of different pH. b) Double layer capacitance found before (dark red) and after (bright red) 24 h at 10 mA cm^−2^ at different pH values in KOH electrolytes. c) Chrono‐potentiometry of Ni−Fe−S/Ni foam at 10 mA cm^−2^ operated in NaOH electrolytes of different pH. For pH 13 data collection stopped after 10 h, but it was confirmed water electrolysis continued for the full 24 h. d) Double layer capacitance found before (dark green) and after (bright green) 24 h at 10 mA cm^−2^ at different pH values in NaOH electrolytes. e) Chrono‐potentiometry of Ni−Fe−S/Ni foam at 10 mA cm^−2^ operated in LiOH electrolytes at pH 13. f) Double layer capacitance found before (dark purple) and after (bright purple) 24 h at 10 mA cm^−2^ in pH 13 LiOH electrolytes. In all figures the pH 13+S samples contain 0.45 M M_2_SO_4_ to maintain a 1 M M^+^ concentration.

The observation of the material being unstable, without significantly changing the OER performance led us to believe that the S leaching behaves similar to the Mo leaching observed in previous works.^34^, ^38^ Likewise, we chose to study whether this leaching is dependent on the electrolyte. In Figure 6 the effect of pH as well as cation is studied. As can be seen activity of these materials is mostly independent on the cation used. The pH expectedly influences the activity: higher pH means higher activity. To exclude this being an ion density effect we also tested the materials in 0.1 M MOH with 0.45 M M_2_SO_4_ electrolytes, maintaining 1 M of M^+^ and though these samples perform better, as could be expected, it does not bring them to the level of those operated in 1 M MOH. It is well studied that the OH^−^ concentration improves the OER, since it reacts via 4 OH^−^→2 H_2_O+O_2_+4 e^−^.^1^ Furthermore, employing M_2_SO_4_ allowed us to study if SO_4_^2−^ could have a stabilizing function similar to S^2−^.^19^

In Figure 6 the change in double layer capacitance is shown as well. The capacitance is often used as a measure of surface area. We opt, however, to use it as a measure of surface change, as we already found that changing surface composition has a large impact as well.^38^ Whereas the activity was not visibly influenced the change in capacitance is. First of all, we see a significant increase in capacitance in all samples, which is in the order of a tenfold increase. However, it can clearly be observed that the final capacitance found in KOH is significantly lower than the one found for NaOH and LiOH. Especially at pH 14 the difference in K and Na containing electrolytes is clear. Interestingly, at pH 14.8 the materials end up at a similar value of 1.57 mF (KOH) and 1.83 mF (NaOH), despite the vast difference in starting capacitance (0.16 mF for KOH, 0.93 mF for NaOH). This strongly suggests that pH and cation have a significantly stronger effect on the catalyst surface than the starting point for said surface.

As was the case with Ni−Mo HER electro‐catalysts, this observation of a changing catalyst surface led us to suspect that leaching occurs. In Table S2 we have listed the values found for Ni, Fe and S after 24 h of catalysis. Interestingly, for the catalysts operated at pH 14.8 a metallic plating was found on the Au counter electrode. This plating was dissolved and measured separately, as shown in Table S2. It was found to mostly consist of Fe and S with traces of Ni. Unfortunately, the pH 14.8 KOH electrolyte crystallized upon preparation for ICP‐AES, resulting in it being unmeasurable by ICP‐AES. Assuming the ratio of plating on Au and solutes in the electrolyte are similar we estimated the values, as shown in Table S2. We find that, in line with the capacitance change, in KOH leaching occurs least in the order of K<Li<Na at pH 13. The trend of leaching being K < Na holds for pH 14 as well. At pH 14.8 there is more leaching into KOH than is into NaOH, which is again in line with the capacitance. The trend of pH with leaching is the similar as the one observed with the capacitance: pH 14.8 NaOH being lowest, though it should be noted that the starting capacitance for this material was significantly higher than most samples. This is followed by pH 14 KOH being low, as is seen in the capacitance as well. Going to lower pH values for KOH resulted in increased leaching. Going to even lower values than reported in Figure 6 and Table S2, namely pH 11, resulted in the Ni−Fe−S/Ni foam being too unstable and the ‘acidity’ of the solution resulted in the entire exposed material, Ni foam included, to be dissolved, and subsequently precipitated as a slimy green solid. Almost no Fe (0.012 μmol) was found in the solution, while relatively high amounts of Ni (0.215 μmol) remained. No comments can be made on S as 0.5 M K_2_SO_4_ was present to maintain electrolyte conductivity. Both the Ni, nor Fe, amounts in solution were high enough to account for the entire material to be dissolved, and thus the precipitation contains both, likely being the hydroxides of both.

Interestingly, when no current is applied while the Ni−Fe−S catalyst is in the electrolyte leaching is approximately double for all three elements present after 24 h. Similarly, when the sample is submerged for 1 week, the leaching is higher for all elements (compared to Table S1) except for Mo, which comes from the Ni−Mo sample also present in this experiment. Mo leaching being increased slightly by the application of current was already found before and thus in line with previous results.^38^ This observation strongly suggests that the application of an oxidative potential on the system suppresses the leaching. The expected modes of leaching for Ni and Fe are oxidative (M→M^x+^+x e^−^). This leaching mode is likely why it destabilized at pH 11, and subsequently precipitated as hydroxides. At higher pH values it is well known from corrosion studies that passivatingxide/hydroxide layers (MOOH, M(OH)_2_) are formed.^39^, ^40^, ^41^ S leaching is likely to occur via H_2_O (S+2 H_2_O→S^2−^+2 OH^−^) and is reported to be first order in H^+^ (effectively present as H_2_O at high pH).^42^ This explains the observation that leaching of S is reduced when an oxidative potential is applied (as S+2 e^−^→S^2−^ is a reduction) and why lower leaching is found for KOH at pH 14 compared to pH 13. It does not, however, explain the higher leaching at pH 14.8 in KOH.

To explore if the observed values are a result of possible differences in the initial concentration of S in the materials SEM‐EDX was employed both prior to and after the 24 h electrocatalysis. As can be seen, for all 3 electrolytes, KOH, NaOH, and LiOH (Figures S5‐S7, respectively), the surface morphology doesn't change significantly on the micrometer scale.

Larger magnifications were not explored due to significant charge build‐up if the electron beam was focused further, which posed a risk to damage the SEM. Interestingly, when comparing EDX values before and after catalysis we observe a clear trend: the Ni/S ratio decreases. This means, that despite the clear leaching of S, the surface concentration of said S increases. Similar to Ni−Mo, this suggests we have surface segregation resulting in higher S surface concentrations. This is again a clear sign that electro‐catalysts are indeed not static under operation conditions. Furthermore, we find K and Na on the samples after catalysis, where the concentration increases with pH and M^+^ concentration in the solution. This is not a result of flushing; differences in flushing time with deionized water after catalysis does not change the observed surface concentration. It is also worth to note that before catalysis rather reproducible values of Ni (70.2±4.1 at%) and S (25.9±3.0 at%) are found, whereas Fe inclusion nearly has an error of 100 % (3.9±3.6 at%). It has to be noted that all the samples reported in Table S3 have been made at the same time in a single vessel. In other words, the treatment was constant. Likely Fe in the solution aids the formation of roughness as was already described earlier. It is not included in the material itself it seems but sooner present as a residue after synthesis, giving a wide spread in its atomic contribution to the material. Indeed, with EDX mapping we find that Fe is mostly present in the particulate matter which is, for example, seen in Figure S6c and Figure S7a.

## 3 Conclusions

We have identified that the high activity of Ni−Fe−S stems from two different effects: synergy between Ni and S and a surface area increase caused by the addition of Fe. High performance stability was found for these materials at a set current and they operate stably for the course of a week at 1.55 V and 10 mA/cm^2^. The system is, however, more sensitive to intermittency in potential and cycling shows a large change in performance after the first few cycles, after which the change is slowed down significantly.

Detailed studies into the Ni−Fe−S material showed that S leaches from the material and segregates towards the surface to result in a surface of Ni_60_S_40_ compared to the Ni_72_S_28_ ratio found beforehand. Fe was found to function mostly to increase the surface area was seems to be not included into the material, but only sometimes present as particles on top of the material, which in most cases are no longer found after catalysis. Changes in capacitance and leaching of S show the same trends, though could not be directly linked, showing that for Ni−Fe−S electrodes surface composition is also a significant contributor to the capacitance value.

The pH of the electrolyte played a significant role in the stability of Ni−Fe−S. Too low pH values result in the destabilization of the metal hydroxide passivating layers. Furthermore, S leaching is influenced by acidity as well and oxidative potentials stabilized the S in the electrodes compared to electrodes being immersed in the electrolyte without applied potential. The cation of the electrolyte was found in the material after operation, where Na is present is significantly larger numbers than K.

At continuous operation at 10 mA/cm^2^ the Ni−Fe−S electrodes were found to be most stable in 1 M KOH solutions of all tested electrolytes. When higher potential values were applied (e. g. via a solar module, Figures S8–S10) the window of stable operation seems to shift towards lower pH.

## Experimental Section

### Chemicals and Materials

All materials were used as received without further purification. NiSO_4_ ⋅ 6H_2_O (ReagentPlus, >99 % pure), FeSO_4_ ⋅ 7H_2_O (ReagentPlus, >99 % pure), NaMoO_4_ ⋅ 2H_2_O (ACS reagent, >99 % pure), NaOH (99.99 % trace metals, semiconductor grade), KOH (ACS reagent, >85 % pure, ca. 15 % H_2_O), Thioacetamide (ACS reagent, >99 % pure), NaOH (99.99 %, trace metals, semiconductor grade), LiOH ⋅ H_2_O (99.95 %, trace metals), Na_2_SO_4_ (ACS reagent, ≥99.0 %), K_2_SO_4_ (ReagentPlus, ≥99.0 %), Li_2_SO_4_ ⋅ H_2_O (BioUltra, ≥99.0 %) and Na_3_C_6_H_5_O_7_ ⋅ 2H_2_O (sodium citrate, ACS reagent, >99 % pure) were received from Sigma Aldrich. NH_3_ 28–30 % (ACS reagent, ph. Eur. for analysis) was obtained from Emsure. In all experiments deionized water was used.

### Ni Foam Preparation

Ni foams were shaped into keyhole shapes (S.I. Figure S.11) using a punch with a 2 cm×0.5 cm lip and a 1 cm^2^ circle. Epoxy (Loctite EA 3425) was used to cover the lower part of the lip to have a set area of the sample exposed. Application was done in two steps to ensure that the pores were filled with epoxy as well to prevent filling by capillary forces. They were then cleaned by sonication in 1 : 1 : 1 demiwater:ethanol:acetone, followed by sonication in 0.1 M KOH, followed by sonication in water. Each sonication step took 15 min.

### Ni‐Mo Electrodeposition

The foams were fixed in a three‐electrode cell (S.I. Figure S.12) for electro‐deposition. Electro‐deposition was performed galvano‐statically using an Ivium Compactstat at a current of −100 mA for 1200 s while stirring at 400 rpm. As a counter electrode a Pt mesh (Mateck, 99.9+%) was used and as a reference electrode a 3 M Ag/AgCl electrode (BASi) was used. The plating bath used contained 0.3 M NiSO_4_, 0.2 M Na_2_MoO_4_&0.3 M Na_3_C_6_H_5_O_7_ in 100 mL demineralized water. To this, 10 mL NH_3_ was added to obtain a pH of 9.2. First, the metals were dissolved in water through stirring, and then NH_3_ was added to adjust the pH. Prior to the syntheses, the baths were purged with Ar for 15 min, and a gentle Ar flow was kept over the solution during electro‐deposition.

### Ni‐Fe‐S Hydrothermal Synthesis

A 150 mL, Teflon‐lined autoclave was filled with 40 mL of solution containing 210 mM thioacetamide, 10 mM FeSO_4_ ⋅ 6H_2_O and a Ni foam were heated to 180 °C in an oven. This was left there for 5 h and then cooled slowly in air before being opened.

### Electrochemistry

100 mL 1 M KOH electrolyte is loaded in a three‐electrode electrochemical cell then deoxygenated for at least 15 min by a flow of 20 mL/min of Ar (5.0 quality). Samples are immersed in the solution just past the beginning of the epoxy layer as a working electrode. Depending on the experiment, the cell is loaded as a two‐electrode cell (with the reference electrode compartment closed off) with another Ni foam, or a Pt mesh electrode as counter electrode. Optimization of Ni−Fe−S was done in a three‐electrode configuration with a 3 M KCl Ag/AgCl electrode (BASi, −1.033 V vs NHE at pH 14). Chronoamperometry measurements are performed at 10 mA/cm^2^ (−10 mA/cm^2^ in case the cathode was studied in a three‐electrode configuration) with steps of 1 s with static electrolytes (no bubbling, no stirring). Gas Chromatography analysis was done with a H‐cell with a Nafion perfluorinated membrane (Nafion 117, 0.007 inch thick, Sigma Aldrich) loaded with the electrodes and 1 M KOH (S.I. Figure S.13). This was purged with 2 mL/min N_2_ (5.0), 0.1 mL/min Kr (5.0) on the O_2_ side and 2 mL/min Ar (5.0), 0.1 mL/min Kr on the H_2_ side. This was bubbled past the samples through a glass frit. The O_2_ electrode was the WE, the H_2_ electrode was the CE/RE. A current of 10 mA/cm^2^ was maintained for 24 h. GC was obtained using Global Analyzer Solutions Compact GC 4.0 from Interscience with separate channels for H_2_ and O_2_. H_2_ was analyzed via a 75 μL sample loop injecting into a 5 m ⋅ 0.53 mm MXT−Q‐bond then a 10 m ⋅ 0.53 MXT‐Msieve column and detected on a TCD. O_2_ was injected via a 50 μL loop through different columns of the same type and analyzed on a separate TCD. An injection was done each minute, with about 10 s delay between each injection. For each injection Kr was used as an internal standard. Ni−Fe−S stability tests were done in 6 M OH^−^ solutions, 1 M OH^−^ solutions, and 0.1 M OH^−^ solutions. The tests with SO_4_^2−^ were done in 0.1 M MOH with 0.45 M M_2_SO_4_. One test, in 0.001 M KOH with 0.5 M K_2_SO_4_ was done as well, but pH 11 was found to be too low and the sample was fully digested, resulting in slimy green NiO precipitation. At pH 14 the tests were done as follows, with all voltages vs Ag/AgCl: 5 CV's from 0 V to −0.2 V and back for 3 cycles at 100, 200, 300, 400, and 500 mV/s, followed by linear sweep voltammetry from 0.23 V to 0.53 V. Then chrono‐potentiometry was performed at 10 mA/cm^2^ for 86400 s. Then the linear sweep was repeated, followed by a repeat of the CV's. At other pH values the same methodology was used, except that the used voltages were adjusted for the pH according to −0.059 V/pH.

### Characterization

X‐ray diffraction (XRD) was measured using a Bruker D2 Phaser instrument with a cobalt anode. Scans were taken from 30–80 °2θ with 0.02 °2θ steps measuring 1 s/step while rotating at 15 Hz. Scanning electron microscopy with energy dispersive X‐ray spectroscopy (SEM‐EDX) was performed on a FEI Helios nanolab 600 DualBeam with an Oxford instruments Silicon Drift Detector X‐Max energy dispersive spectroscope. SEM imaging and EDX mapping was performed with an electron beam of 15 kV and 0.8 nA. Inductively coupled plasma atomic emission spectroscopy (ICP‐AES) was performed using an Optima 8300 instrument from Perkin Elmer and an average of three samples was used. Electrodeposited samples were dissolved in 10 mL 2 % HNO_3_ before oxidation. These were diluted with 2 % HNO_3_ to achieve optimal measurement ranges. Electrolytes were decreased in pH by adding 1 mL 65 % HNO_3_ per 10 mL electrolyte, resulting in ca. 2 % HNO_3_. Ni (231.604 nm), Fe (259.941 nm), S (182.034 nm) and Mo (202.095 nm, 203.909 nm) were then measured.

## Conflict of interest

The authors declare no conflict of interest.

## Supporting information

As a service to our authors and readers, this journal provides supporting information supplied by the authors. Such materials are peer reviewed and may be re‐organized for online delivery, but are not copy‐edited or typeset. Technical support issues arising from supporting information (other than missing files) should be addressed to the authors.

Supplementary

Supplementary
