# Supplementary material for: Basicity and Electrolyte Composition Dependent Stability of Ni‐Fe‐S and Ni‐Mo Electrodes during Water Splitting
Source: Chemphyschem. 2020 Feb 11;21(6):518–24. doi: 10.1002/cphc.201901219 (PMC7155041; doi:10.1002/cphc.201901219)
Supplement: Supplementary file 2 — Supplementary [file CPHC-21-518-s002.pdf]

CHEMPHYSCHEM

Supporting Information

Basicity and Electrolyte Composition Dependent Stability of Ni-Fe-S and Ni-Mo Electrodes during Water Splitting

Jochem H. J. Wijten, Iván Garcia-Torregrosa, Eva A. Dijkman, and Bert M. Weckhuysen*© 2020 The Authors. Published by Wiley-VCH Verlag GmbH & Co. KGaA.
This is an open access article under the terms of the Creative Commons Attribution License, which permits use, distribution and reproduction in any medium, provided the original work is properly cited.

Supporting Information

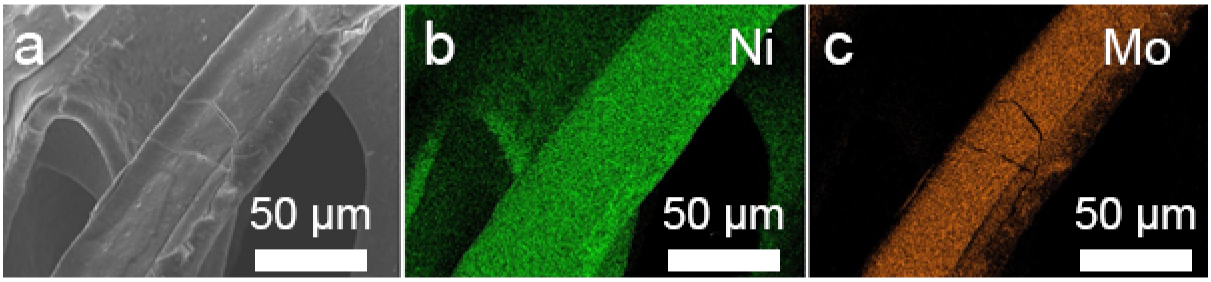

Figure S1: *a) SEM-EDX micrograph of Ni-Mo. b) Ni distribution over the sample. c) Mo distribution over the sample.*

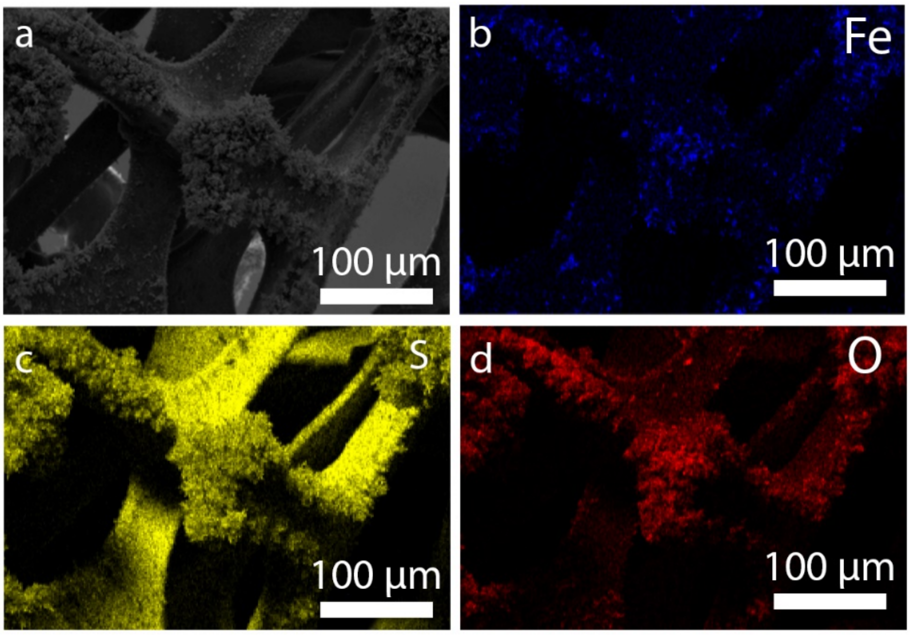

Figure S2: *a) SEM-EDX micrograph of Ni-Fe-S. b) Fe distribution over the sample. c) S distribution over the sample. d) O distribution over the sample.*

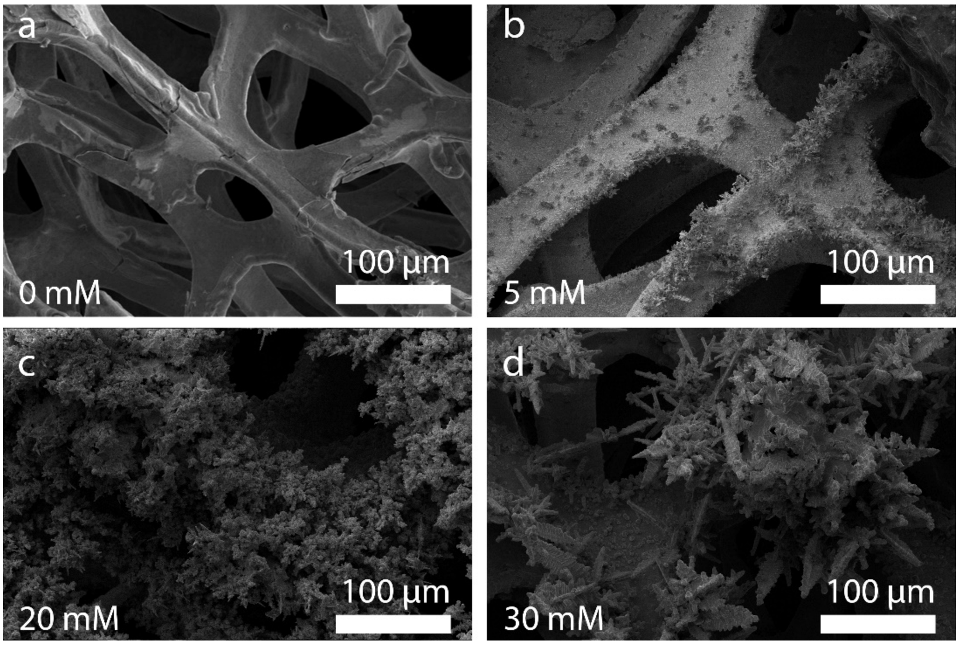

Figure S3: *Effect of $FeSO_4$ concentration, a) 0 mM, b) 5 mM, c) 20 mM, and d) 30 mM, on the morphology of Ni-Fe-S.*

***Table S1**: Amount of µmol leached into 1 M NaOH electrolytes as found by ICP-AES for Ni, Fe, S and Mo for various combinations of electrodes after 16 h of operation (168 h for Ni-Mo vs Ni-Fe-S). Values were measured as mg/kg and corrected to take into account the 1.1x dilution with HNO_3 and the total volume of 100 mL.*

HER vs OER	Ni (µmol leached)	Fe (µmol leached)	S (µmol leached)	Mo (µmol leached)
Ni Vs Pt (16 h)	0.084	0.003	-0.050	0.069
Pt vs Ni (16 h)	0.084	0.012	0.048	0.017
Pt vs Ni-Fe-S (16 h)	0.073	0.004	5.999	0.010
Ni-Mo vs Pt (16 h)	0.081	0.000	-0.102	3.189
Ni-Mo vs Ni-Fe-S (168 h)	0.064	0.035	12.319	2.724

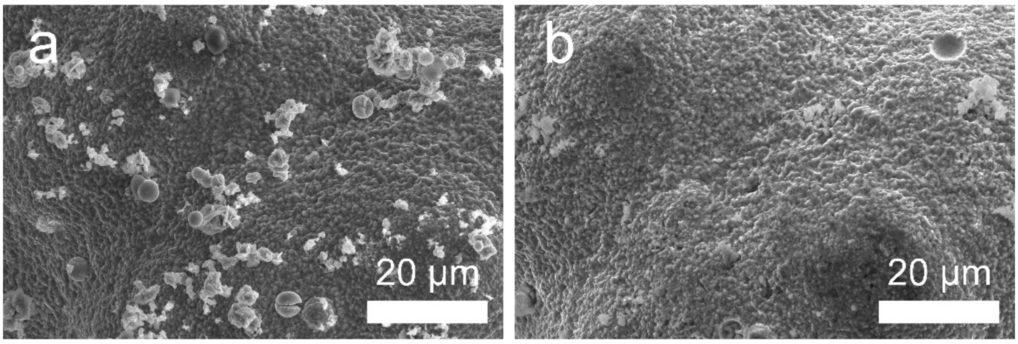

***Figure S4**: SEM images of Ni-Fe-S/Ni foam a) before and b) after 1 week of continuous operation at 10 mA/cm^2 in 1 M KOH vs Ni-Mo/Ni foam.*

***Table S2**: Amount of Ni, Fe, S and Mo leached into the electrolyte as detected by ICP-AES after electrocatalysis for 24 hours, unless noted otherwise. [a] These values are estimated based on the ratio of plating and electrolyte concentration of NaOH at ph 14.8. [b] In this electrolyte both a Ni-Fe-S/Ni foam and Ni-Mo/Ni foam sample were present. Values were measured as mg/kg and corrected to take into account the 1.1x dilution with HNO_3 and the total volume of 100 mL.*

	Ni (µmol leached)	Fe (µmol leached)	S (µmol leached)	Mo (µmol leached)
KOH pH 13	0.026	0.113	7.104	-
KOH pH 14	0.019	0.134	4.226	-
KOH pH 14.8[a]	*0.046*	*0.850*	*16.336*	-
KOH pH 14.8 plating on Au	0.180	9.430	18.684	-
KOH pH 14, 24 h, no current	0.030	0.330	8.918	-
KOH pH 14, 1 week, no current[b]	0.103	0.306	31.209	2.345
NaOH pH 13	0.012	0.133	9.730	-
NaOH pH 14	0.008	0.215	17.264	-
NaOH pH 14.8	0.018	0.345	3.141	-
NaOH pH 14.8 plating on Au	0.070	3.826	3.592	-
LiOH pH 13	0.007	0.072	8.38	-

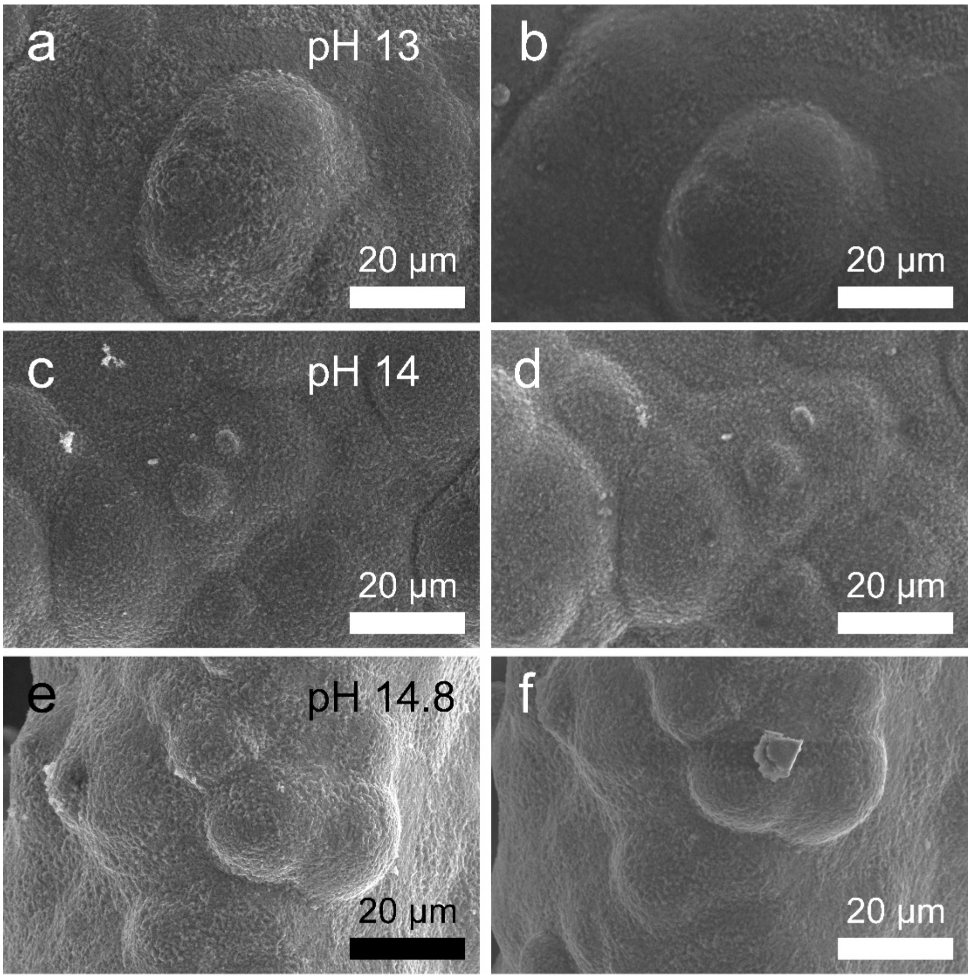

Figure S5: *SEM micrographs of Ni-Fe-S/Ni foam of a) fresh and b) spent in pH 13 KOH for 24 h, c) fresh and d) spent in pH 14 KOH for 24 h, and e) fresh and f) spent in pH 14.8 KOH for 24 h.*

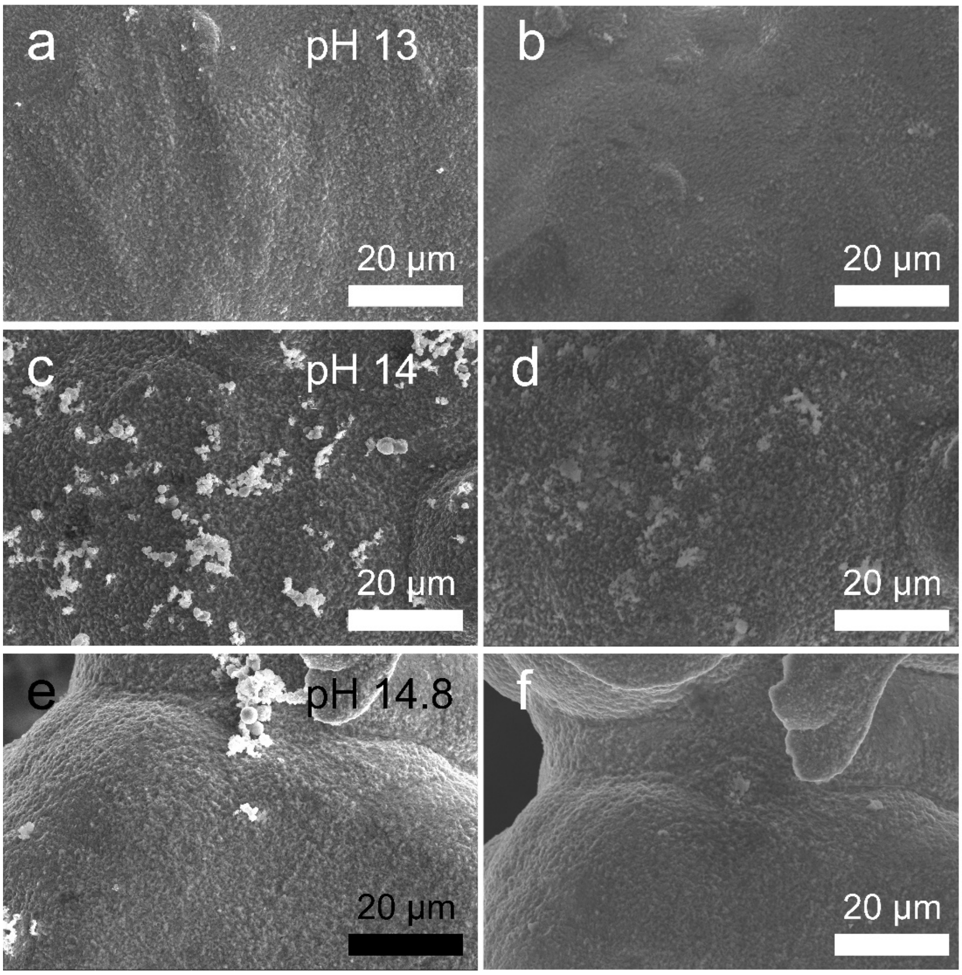

Figure S6: *SEM micrographs of Ni-Fe-S/Ni foam of a) fresh and b) spent in pH 13 NaOH for 24 h, c) fresh and d) spent in pH 14 NaOH for 24 h, and e) fresh and f) spent in pH 14.8 NaOH for 24 h.*

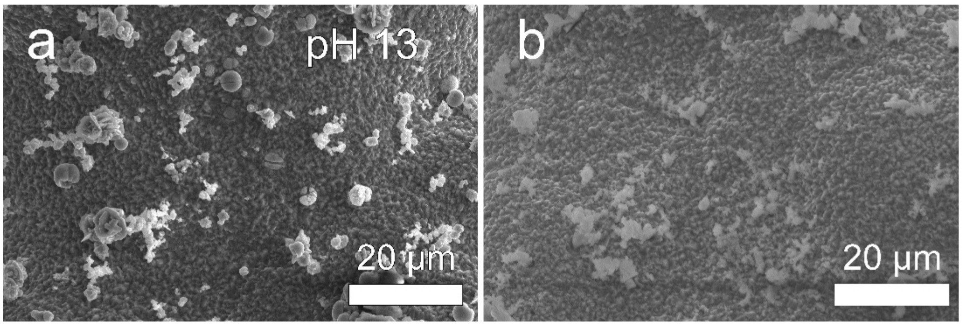

Figure S7: *SEM micrographs of Ni-Fe-S/Ni foam of a) fresh and b) spent in pH 13 LiOH for 24 h.*

***Table S3**: Atomic ratio's found by EDX of Ni, Fe, S, K and Na before (normal) and after (bold) catalysis at 10 mA/cm^2 for 24 h, unless noted otherwise. Other elements (adventitious C, O) were observed but are not included in the calculation. Li at% values were not determined since it emits X-rays of too low energy to be quantified. Values were measured as mg/kg and corrected to take into account the 1.1x dilution with HNO_3 and the total volume of 100 mL.*

	Ni (at%)	Fe (at%)	S (at%)	K (at%)	Na (at%)
KOH pH 13	65.0	8.1	26.9	-	-
	**61.6**	**2.9**	**35.4**	**0.1**	**-**
KOH pH 13 + S	72.1	0.1	27.8	-	-
	**60.6**	**0.1**	**38.9**	**0.5**	**-**
KOH pH 14	73.5	0.1	26.4	-	-
	**62.0**	**0.1**	**37.0**	**0.9**	**-**
KOH pH 14.8	73.6	0.2	26.2	-	-
	**63.0**	**0.2**	**34.3**	**2.6**	**-**
KOH pH 14, 24 h,	66.5	7.7	25.8	-	-
no current	**53.3**	**9.2**	**34.7**	**2.8**	**-**
KOH pH 14, 1	64.5	6.7	28.9	-	-
week, no current	**67.8**	**5.4**	**25.0**	**1.8**	**-**
NaOH pH 13	73.2	0.0	26.8	-	-
	**57.5**	**0.1**	**42.2**	**-**	**0.2**
NaOH pH 13 + S	75.7	7.9	16.5	-	-
	**61.2**	**1.2**	**34.4**	**-**	**3.2**
NaOH pH 14	72.0	3.6	24.4	-	-
	**58.1**	**0.7**	**36.5**	**-**	**4.7**
NaOH pH 14.8	71.3	1.4	27.3	-	-
	**57.7**	**0.3**	**35.2**	**-**	**6.7**
LiOH pH 13	63.1	9.2	27.7	-	-
	**55.6**	**2.8**	**41.6**	**-**	**-**
LiOH pH 13 + S	72.2	1.9	25.9	-	-
	**60.2**	**2.4**	**37.4**	**-**	**-**

Solar Driven Water Splitting Demonstration

Ni-Mo forms a slightly darker material on the Ni foam, while Ni-Fe-S forms a nearly black compound. On one hand, these can be combined in a controlled system and perform exceptionally at 10 mA/cm^2 and stay stable on 1.55 V for at least one week. On the other hand, we also present a system driven solely by a solar module consisting of four solar cells. These deliver 2.1 V and 1 A under 1 sun (1.5 AM) illumination. To accommodate these values, we tested the materials in 0.1 M KOH, since at 2.1 V Ni-Fe-S oxidizes to Ni^{2+} and subsequently precipitates as NiO in a 1 M hydroxide solutions. From this resulted that 10 cm^2 is the optimal electrode size to accommodate the full current (Figure S8).

A movie and snapshots of a movie (Figure S9), made of the demonstration setup (Figure S10) can be found in the Supporting Information. For practical purposes an electrode size of 15 cm^2 is chosen to relieve electrochemical strain on the electrodes, in case the light intensity is higher than 1.5 AM. It can be observed that indeed extensive bubble formation occurs when the setup is running in the sun. Furthermore, as is expected, the amount of bubbles formed at the HER side are larger than those at OER. Interestingly the bubbles formed at the HER are visibly smaller than the bubbles at OER, suggesting that H_2 releases from the material more readily than O_2, though more research is needed to explore if this is a result from the difference in surface morphology of the catalysts or a result of the different physical properties of the gasses.

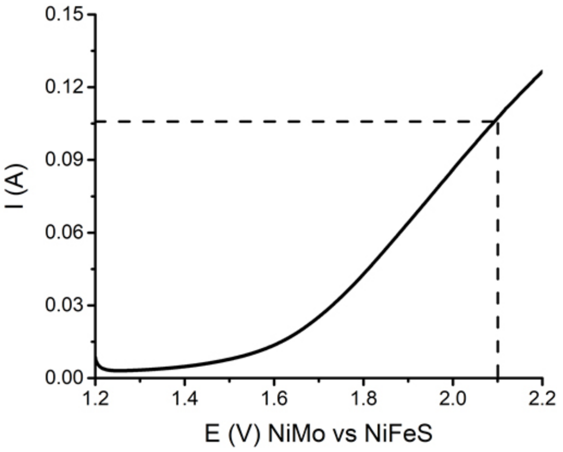

Figure S8: *Ni-Fe-S vs Ni-Mo linear sweep with each electrode consisting of 1 cm^2 Ni foam. Dotted lines show the measured 2.1 V of the solar module resulting in 0.105 A/cm^2.*

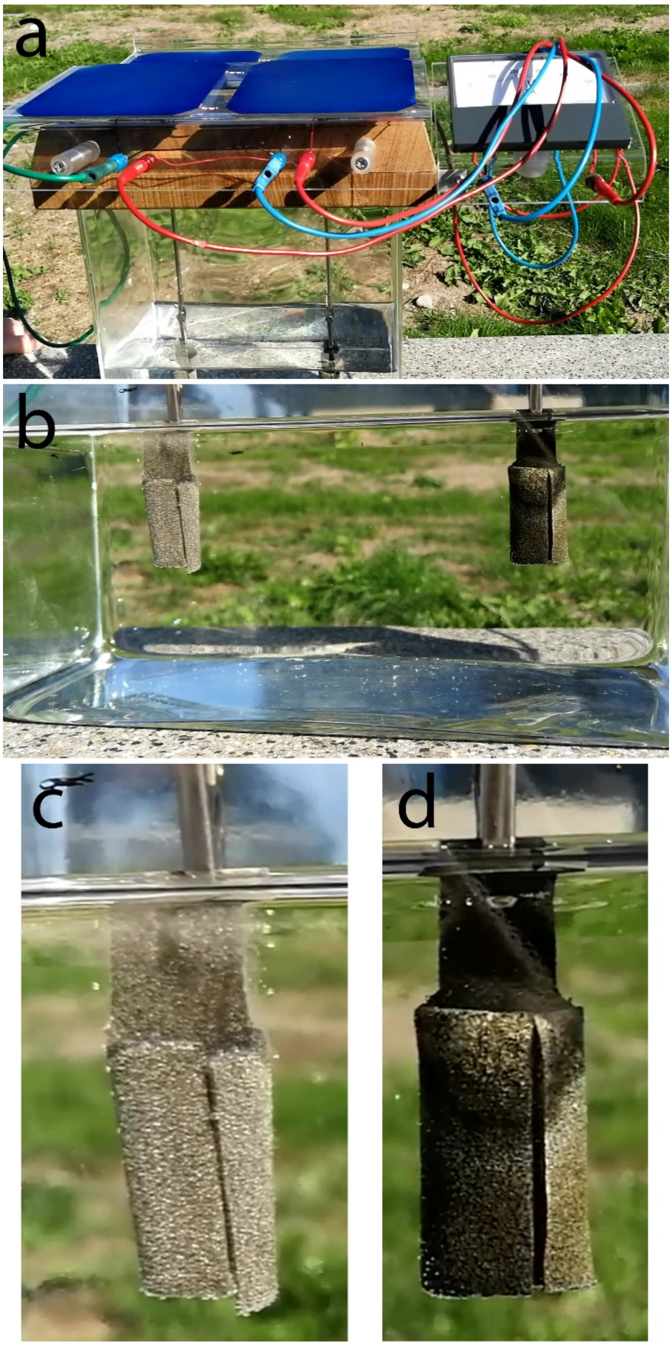

***Figure S9**: Snapshots of a movie made while running the solar cell driven demonstration setup outside in the sun. a) Overview showing the solar cells connected to the electrodes. b) Picture of the electrodes with Ni-Mo/Ni foam on the left performing HER and Ni-Fe-S/Ni foam on the right performing OER. c) Zoomed-in picture of the Ni-Mo/Ni foam HER electrode. d) Zoomed-in picture of the Ni-Fe-S/Ni foam OER electrode. Water reflections cause the crocodile clip to seem submerged but it was just above the electrolyte level.*

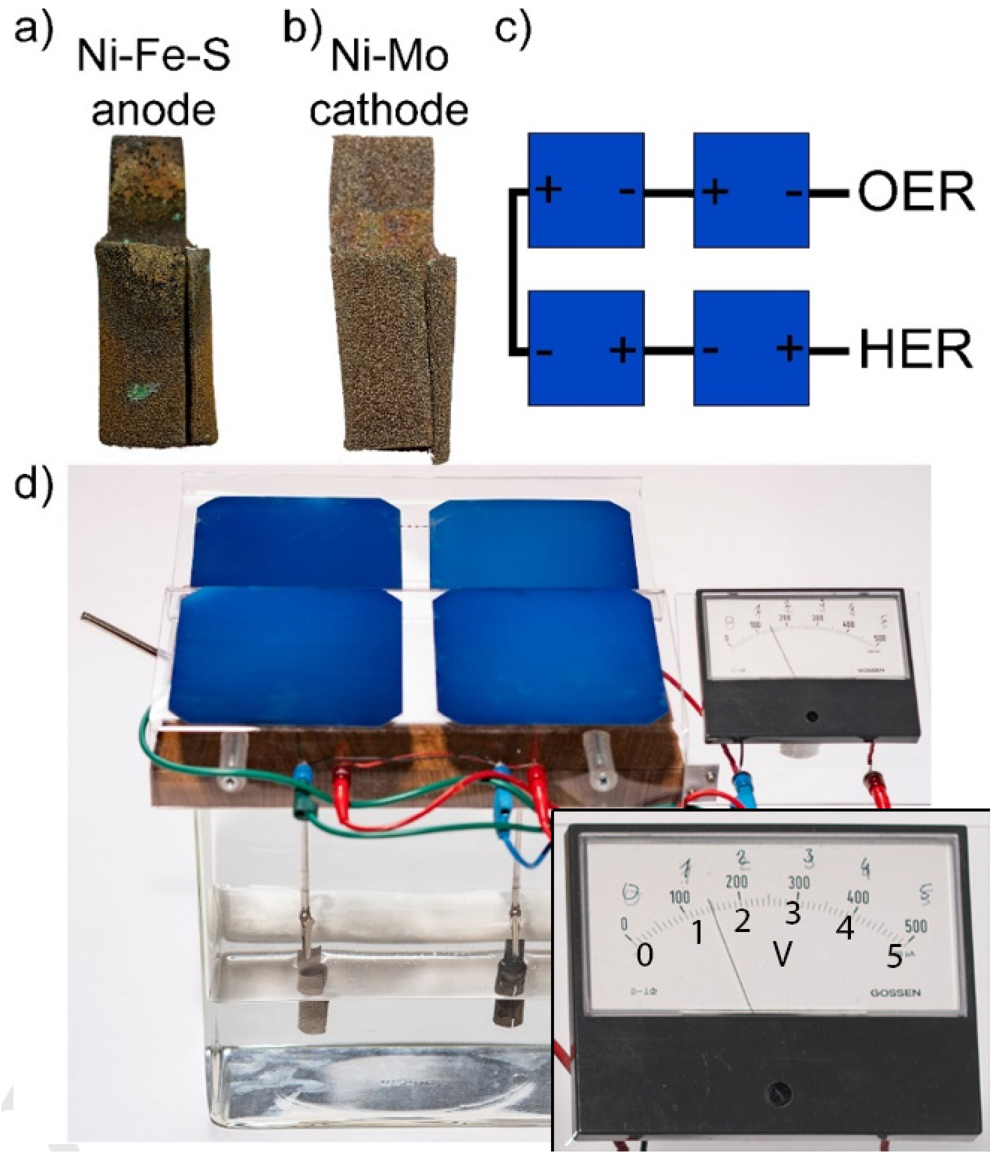

Figure S10: *a) Photograph of a 15 cm² Ni-Fe-S electrode. b) Photograph of a 15 cm² Ni-Mo electrode. c) Schematic representation of the electrical connection of the four solar panels in series and their connection to the HER and OER reactions. d) Photograph of the demonstration setup outside with four solar panels mounted on top of a wooden top. The electrolyte is 0.1 M KOH. There are furthermore connections to a demonstrative voltmeter (repurposed from an amperemeter) showing 2.1 V potential under sunlight and 1.5 V under artificial TL light (inset picture). The values are shown with overlaid text to clarify the reflective numbers. These values were confirmed with a digital multimeter.*

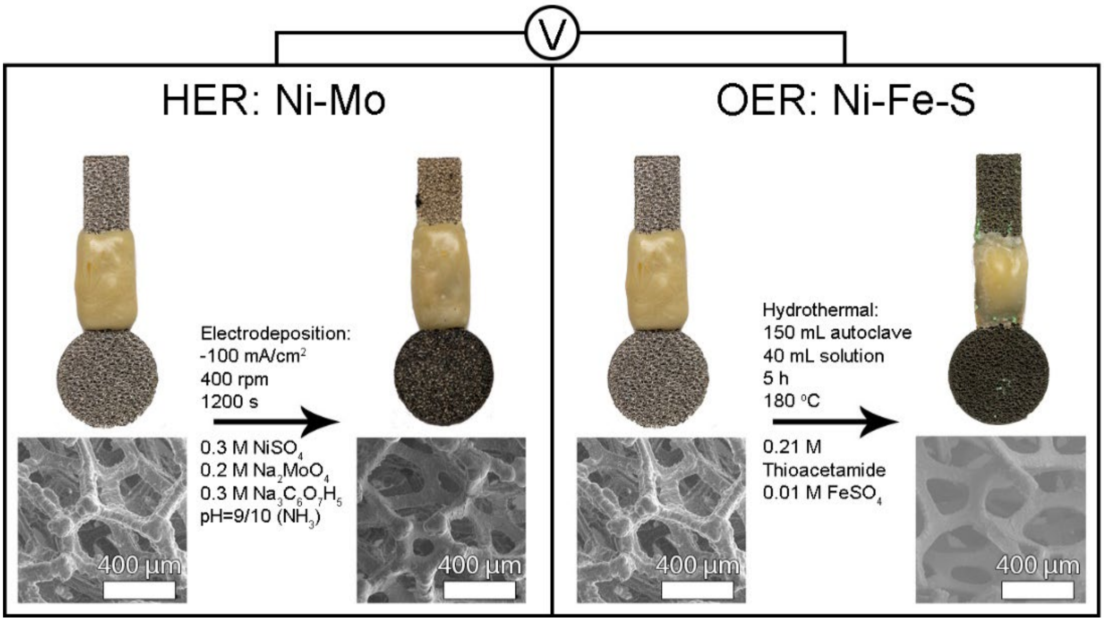

***Figure S11**: On the left are photographs and SEM images of Ni foams before and after electrodeposition of Ni-Mo. On the right are photographs and SEM images of Ni foams before and after hydrothermal synthesis of Ni-Fe-S.*

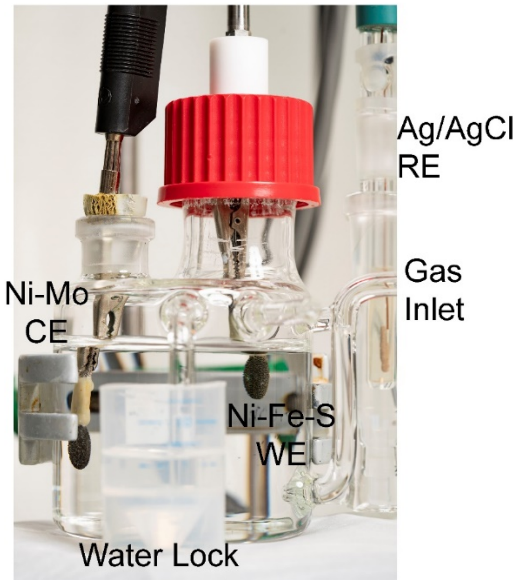

***Figure S12**: Photograph of the three-electrode electrochemical cell used in this work. There are two gas inlets: one for bubbling before an experiment and one for a constant Ar flow during the experiment. A gas outlet through a water lock prevents ambient air from infiltrating the cell. The electrodes are attached as follows: Ni-Fe-S anodes are the working electrode, Ni-Mo cathodes are the counter electrode. Ag/AgCl (3 M KCl) is sometimes used as a reference electrode (three-electrode configuration, as shown in the picture). When a two-electrode configuration was used the compartment separated by the Luggin capillary was closed off with a stopper. For photographic purposes the electrolyte compartment was filled higher to prevent reflections, during the experiments it was made sure the clips were not in contact with the electrolyte. Furthermore, they were covered in Teflon tape (see also Figure S13) to prevent any damage or side reactions with the electrolyte that could be caused by capillary forces or aerosols.*

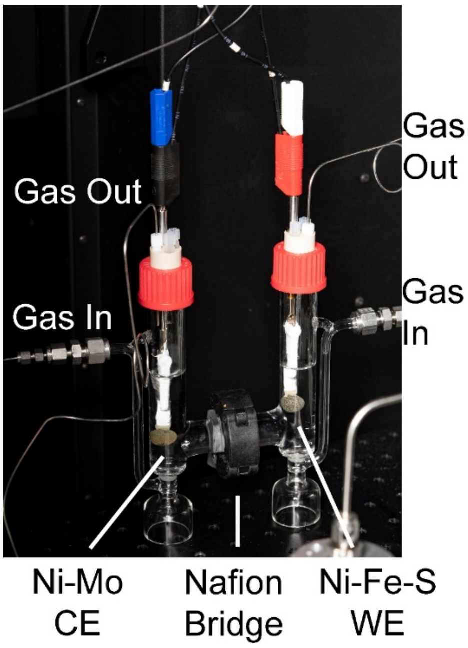

Figure S13: *Photograph of the H-cell used for Faradaic Efficiency measurements. Two compartments are separated by a Nafion proton exchange membrane so that* H_2 *and* O_2 *could be measured by separate channels in the GC. There are gas inlets through glass frits flowing 20:1, 2.1 mL/min flows of Ar/Kr (Cathode side) or* N_2*/Kr (Anode side). No Ar was used on the anode side due to the overlap with* O_2 *in the installed columns.*
